# A Pragmatic Framework for Federated Learning Risk and Governance in Academic Medical Centers

**DOI:** 10.2196/80022

**Published:** 2026-02-27

**Authors:** Daniel Bottomly, Bridget Barnes, Kuli Mavuwa, Nikki Lee, Holger R Roth, Chester Chen, Shannon K McWeeney

**Affiliations:** 1 OHSU Knight Cancer Institute, 3485 S Bond Ave, Portland, OR, 97239, United States, 1 503-494-8311; 2 Office of Information, Privacy and Security, Information Technology Group, Oregon Health & Science University, Portland, OR, United States; 3 NVIDIA, Santa Clara, CA, United States

**Keywords:** federated learning, academic medical centers, governance, security and privacy, artificial intelligence

## Abstract

With the rapid development of artificial intelligence (AI), particularly large language models, there is growing interest in adopting AI approaches within academic medical centers (AMCs). However, the vast amounts of data required for AI and the sensitive nature of medical information pose significant challenges to developing high-performing models at individual institutions. Furthermore, recent changes in government funding priorities may result in the decentralization of biomedical data repositories that risk creating significant barriers to effective data sharing and robust model development. This has generated significant interest in federated learning (FL), which enables collaborative model training without transferring data between institutions, thereby enhancing the protection of proprietary and sensitive information. While FL offers a crucial pathway to enable multi-institutional AI development while maintaining data privacy, it also exposes AMCs to novel governance, security, and operational risks that are not fully addressed by existing procedures. In response, this manuscript provides a perspective grounded in both leading international standards (NIST AI RMF [National Institute of Standards and Technology Artificial Intelligence Risk Management Framework], International Organization for Standardization (ISO) and International Electrotechnical Commission (IEC) 42001) and in the real-world governance experience of AMC leadership. We present a risk differentiation framework, an FL risk matrix, and a set of essential governance artifacts—each mapped to key institutional challenges and reviewed for alignment with core standards but offered as pragmatic, illustrative guides rather than prescriptive checklists. Together, these tools represent a novel resource to support AMC security, privacy, and governance leaders with standards-informed, context-sensitive tools for addressing the evolving risks of FL in biomedical research and clinical environments.

## Introduction

Following the groundbreaking work in artificial intelligence (AI), especially with respect to large language models, there has been an exponential rise in AI model adoption across academic medical centers (AMCs) [1,2] . However, currently, AI governance across AMCs is highly variable [3,4] as is the maturity of information, privacy, and security oversight with respect to AI. Deploying AI effectively in AMCs often necessitates leveraging sensitive patient data for external vendor partnerships or collaborative foundational model training. However, the level of understanding about alternative approaches and perceived risk leads to “over-restrictive” policies that impact data sharing and collaboration. Federated learning (FL), a decentralized approach to training AI or other machine learning (ML) models, has been proposed as a solution for collaboration across AMCs and with industry partners [5]. While FL ensures that sensitive data remains within institutional boundaries and is not transferred between organizations, AMCs must still address significant security and privacy considerations. Even though raw data does not move, model updates exchanged during FL can potentially leak information or be vulnerable to adversarial attacks, and issues of trust and compliance remain critical. The motivation for this work is to provide AMCs with practical guidance and a structured framework for evaluating and mitigating these unique risks, supporting secure and privacy-preserving collaborative AI development in health care settings.

FL is an approach where multiple sites collaborate to jointly train a ML model [6]. Originally, FL was devised as a solution to data privacy issues by not requiring institutions or sites to share their data directly. Benchmark studies have shown its potential effectiveness to bolster the efficacy of model development in biomedical research as reviewed previously [7]. FL can generally be divided into 3 main types [8]: (1) vertical, where the sites share the same samples but have different features, (2) horizontal, where the sites share comparable features but on different samples, and (3) federated transfer learning, which is used when sites have both different samples and different features, allowing them to collaboratively improve models by leveraging transfer learning techniques, even in the absence of significant overlap in data. For the rest of the manuscript, we focus on horizontal FL as that is more likely to be the most common scenario for an AMC. For instance, a horizontal FL use case would be genomics, imaging, and electronic health record data collected from patients with similar diagnoses across different hospitals or catchment areas.

Although, in principle, allowing institutions or sites to control their own data improves privacy, the process of model training using FL has been shown to be susceptible to attacks affecting model performance (training or inference) as well as privacy attacks; for a recent survey, see Rodríguez-Barroso et al [9] and references within. It is important to note that the security and robustness of an FL system depends not only on the security of individual clients but also on the strength of the aggregation protocol and the defenses in place against adversarial behavior. While a compromised client can pose significant risks, properly designed FL systems can mitigate their impact. With regard to the risks if a client is compromised, they include not only the client’s local data but also the data of other clients through model or gradient inversion attacks. A compromised client can also be used to influence the global model through the manipulation of the local data (data poisoning), interference with the local model training (model poisoning), or degradation of the overall training process through so-called Byzantine attacks [10,11]. In addition to the clients, the FL server itself must be protected and monitored, especially for high-risk data. Although no data resides on the server, access to the global model and subsequent updates provides an attacker the means to reconstruct or infer client data. For more information, these attacks and possible mitigations in an FL context have been thoroughly reviewed [12].

Within an AMC from a cybersecurity perspective, a “bad actor” would be an individual with malicious intent toward the data or model. However, we recognize that this is a low-probability scenario. The more likely concern would be at the organizational level when there is a federation with external partners.

Recent efforts to formalize AI governance, including National Institute of Standards and Technology Artificial Intelligence Risk Management Framework (NIST AI RMF) and International Organization for Standardization (ISO) and the International Electrotechnical Commission (IEC; ISO/IEC) 42001, offer foundational structures for risk identification, evaluation, and organizational oversight of advanced AI systems in health care. However, while these standards address key technical and ethical dimensions, they do not fully capture the unique challenges AMCs face when implementing FL initiatives. Specifically, FL introduces cross-institutional accountability gaps, complex role-based risks, new vectors for privacy leakage, and ambiguity in operationalizing risk stratification for projects involving sensitive data or novel model architectures.

For AMCs, these governance gaps manifest in several areas: (1) lack of shared accountability and decision-making models across partnered institutions, (2) difficulties integrating local policy and national or international standards, especially when risk is jointly determined by data sensitivity, model complexity, and evolving clinical context, (3) ambiguity in managing platform-specific risks (eg, role privilege and authorization configuration) and documenting privacy or security practices that span organizations, and (4) insufficient practical guidance for balancing innovation with patient safety, ethical considerations, and regulatory compliance. Our framework aims to address these gaps by combining standards-informed principles with lessons learned from direct AMC experience, offering practical, context-sensitive tools to support cross-functional governance for FL.

As part of this manuscript, we lay out the information, privacy, and security risks for an AMC associated with the use of FL. There are currently a diverse array of FL platforms available (Table S1 in Multimedia Appendix 1). We will illustrate the framework using the NVIDIA FLARE (NVIDIA Federated Learning Application Runtime Environment) [13] platform, as this was the solution used for the first year of the National Cancer Institute FL network prototype [14]. NVIDIA FLARE is NVIDIA’s full-featured open-source FL software development kit [13]. It can support a variety of ML models, including neural networks (colloquially referred to as AI), and is platform-agnostic so that models can be easily migrated to the federated setting. Importantly, it supports the implementation of privacy and security methods like differential privacy (DP) and homomorphic encryption (HE). Although we discuss specific risks related to roles and artifacts for NVIDIA FLARE, the topics and general recommendations can be applied to any FL platform. This information is expected to be relevant to leaders involved in security, privacy, and IT (eg, Chief Information Security Officer [CISO], Chief Privacy Officer, and Chief Information Officer), as well as those involved in data and AI governance at AMCs. Drawing on our experience with the National Cancer Institute FL network initial efforts, we bridge the gap between existing standards and their real-world implementation by providing a practical mapping that has so far been missing.

The development of the proposed FL risk matrix in this manuscript was explicitly guided by international standards and best practices in AI risk management and governance. We combined the flexible, risk-based approach of the NIST AI RMF [15] with the structured, process-driven requirements of ISO/IEC 42001 [16] to create practical tools for oversight in AMC FL contexts.

## Proposed FL Risk Matrix

Our goal was to develop a systematic, transparent approach for managing the complex risks associated with FL in AMCs that leverages established AMC procedures for risk determination—evaluating both data and model risk based on defined criteria (Table 1)—as the critical first step in the review process. This table summarizes critical factors influencing risk: data sensitivity (including regulatory scope), model complexity, and operational context. This was based on both “Map” and “Measure” functions of the NIST AI RMF and the risk assessment procedures in ISO/IEC 42001 Clause 6.1. These standards emphasize identifying, categorizing, and proportionally responding to diverse risk dimensions, including legal and operational impact, which are especially salient in AMC settings. We note that risk determination (using Table 1) is conducted collaboratively by the Artificial Intelligence Governance Committee (AIGC) and the CISO, integrating technical, ethical, and operational expertise to ensure robust, context-sensitive evaluation from the outset. Once risk levels are assigned, the FL matrix (Table 2) directly guides the appropriate level of artificial intelligence governance review (AIGR) and security review (SecR): projects classified as high risk undergo comprehensive, multilevel oversight, while those identified as low risk are eligible for expedited, streamlined review. This design follows the “Manage” function of the NIST AI RMF and ISO/IEC 42001 Clauses 8 and 9, which require that risk controls and performance evaluations are matched proportionately to risk profiles determined in earlier steps. Through this, the matrix ensures that high-risk FL initiatives undergo the strictest oversight, in line with international guidance. To facilitate this review process, we have also identified key artifacts such as essential documentation, authorization files, and privacy configuration files, which are pivotal for transparency, audit readiness, and reliable governance (Textbox 1). These requirements map to the NIST AI RMF’s recommendations for rigorous documentation and continuous oversight and ISO/IEC 42001 Clause 7.5, which details retention of documented information as evidence of compliance and governance efficiency. Together, these tables embody leading principles from NIST AI RMF and ISO/IEC 42001, adapted to the practical realities of AMC FL. They are intended as conceptual and illustrative guides, framing rigorous governance conversations, rather than serving as prescriptive or validated assessment instruments. By aligning review pathways with the specific risk profile of each project, the matrix ensures that institutional resources are efficiently allocated, enabling responsible innovation while maintaining rigorous compliance and data protection standards.

Building on the risk framework, the following sections delve into the specific governance and security mechanisms that ensure context-based, responsible, and trustworthy implementation of FL within AMCs, as well as two illustrative examples.

**Table 1.** Key differentiators between high and low risks for data and models.

Category and factor	High risk	Low risk
Data		
Data sensitivity	PHI^a^, genomic, rare conditions, regulatory considerations (HIPAA^b^, GDPR^c^, EO^d^ 14117)	Aggregated, synthetic, logs, general research data protection(s)
Model		
Model complexity	LLMs^e^, GANs^f^, high-capacity CNNs^g^	Linear models, decision trees
Operational context	Direct impact on patient care, diagnostics, or clinical decision support; real-time or near–real-time use; high stakes for errors, regulatory requirements (FDA SAMD^h^, EU AI^i^ Act)	Administrative, research, or quality improvement tasks; retrospective analysis; minimal patient safety impact

a PHI: protected health information.

b HIPAA: Health Insurance Portability and Accountability Act.

c GDPR: General Data Protection Regulation.

d EO: executive order.

e LLMs: large language models.

f GANs: generative adversarial networks.

g CNNs: convolutional neural networks.

h FDA SAMD: Food and Drug Administration Software as a Medical Device.

i EU AI: European Union’s Artificial Intelligence Act.

**Table 2.** Proposed federated learning risk matrix.

Data risk	Model risk	AIGR^a^^,^^b^	SecR^c^
High	High	Suitability Generalizability Bias/fairness Memorization Robustness to attack	ProjectAdmin and OrgAdmin must be highly trusted and reviewed RBAC^d^ Mandatory HE^e^ and/or DP^f^
High	Low	Memorization Robustness to attack	ProjectAdmin and OrgAdmin must be highly trusted and reviewed RBAC
Low	High	Suitability Generalizability Bias/fairness Robustness to attack	RBAC with additional scrutiny for Leads and OrgAdmins
Low	Low	Expedited	RBAC

a AIGR: artificial intelligence governance review.

b We are separating into AIGR and SecR sections, but in practice, as mentioned in the paper, these activities would be handled jointly.

c SecR: security review.

d RBAC: role-based access control following the least privilege principle.

e HE: homomorphic encryption.

f DP: differential privacy.

Textbox 1. **Relevant artifacts for artificial intelligence (AI) governance review and security review.**

The following are the artifacts for AI governance review and security review:

- Suitability
  - Computational notebooks
  - Model cards
  - Prior publications
- Pretraining evaluation and certification
  - Computational notebooks
- Roles and authorization
  - Role qualification document
  - Authorization.json
- Privacy and security of model updates
  - privacy.json
  - config_fed_server.conf
  - config_fed_client.conf
  - Computational notebooks
    - Provide data for choice of *eps* values if differential privacy is used

## AI Governance Review

Following risk determination for both the models and data, the project advances to the AIGR. Recognizing the critical importance of FL, our approach assumes that robust AI governance structures are in place at AMCs [3,4]. Based on our FL risk matrix, we propose that for any project involving high-risk data and/or high-risk models, AIGR consists of 2 primary steps: (1) assessing model suitability for its intended use and (2) conducting pretraining evaluation and certification for FL. For suitability, comprehensive model documentation (such as Model Cards [17]) is required or, at minimum, evidence that the model architecture and training pipeline have been validated on publicly available or simulated data. It is essential to confirm that the model’s intended use matches its validated context and that security, privacy, and ethical, legal, and social implications are explicitly addressed.

Pretraining evaluation and certification consists of 4 main components: generalizability, fairness and bias, memorization risk, and susceptibility to attack. With regard to generalizability, does the training pipeline maintain consistent performance across all datasets from participating sites? Are there any detectable issues with fairness or bias against certain groups, which can be quantified using several toolkits [18-20]? Note, this is separate from suitability above, which is focused on the concept of the predictive task—here we are addressing specifically the data to be used for this application. How likely is the model to memorize training data, especially rare instances? Memorization or the ability of an AI or ML approach (therefore not unique to FL) to recall specific training examples [21] can be measured after training using, for instance, the exposure metric for natural language data [22] or the *M* score as proposed in the context of medical imaging [23] as well as other metrics now being proposed [24]. We note that memorization can be mitigated using components in FL such as DP [25-28] (as discussed later). Finally, the most challenging assessment would be to determine the susceptibility of a model training pipeline to the wide range of possible attacks. This could be performed via red-teaming endeavors using NVIDIA FLARE’s simulation software.

Once a model is certified and trained using FL, it undergoes posttraining assessments. These would be like the pretraining assessment but would be applied to the final trained model. A model passing these assessments could then be deployed. Monitoring of a deployed model would then proceed as part of normal ML operations [29] best practices. The collaborative involvement of the CISO and AIGC in this phase ensures that both technical and ethical standards are rigorously maintained and that the institution remains compliant with evolving regulatory and industry best practices.

## Security Review

**Figure 1.**
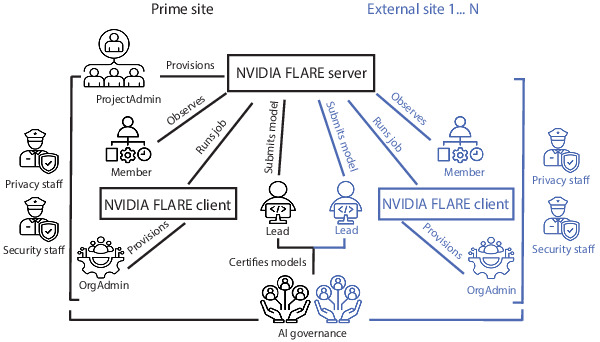
Relationships and responsibilities of entities and roles involved in federated learning with NVIDIA FLARE. In the creation of a federated learning (FL) network, there will be a “prime site“ who provisions the network and provides guidance for other external sites (blue). For this discussion based on the National Cancer Institute F network, we assume the Prime site is also the site that is responsible for the federated model. Outside of the Project Admin role and the Artificial Intelligence Governance Committee (AIGC), each site will have representatives assuming one or more of the NVIDIA FLARE roles. The AIGC should have representatives from the sites and oversee all of the modeling work that occurs as part of the FL network as well as approving the FL framework itself. Each site will have security and privacy officers who will be in charge of ensuring the security of any high-risk data and/or models and compliance with privacy laws, with input from the AIGC. Shown as icons are the different roles in addition to the individuals involved in governance or oversight. Boxes indicate the computational resources such as the NVIDIA FLARE server and clients. Lines indicate the main relationships with accompanying text. AI: artificial intelligence; NVIDIA FLARE: NVIDIA Federated Learning Application Runtime Environment.

The SecR is completed in parallel with the AIGR and focuses on (1) user roles, (2) authorizations, and (3) the privacy and security of model updates. Within the NVIDIA FLARE platform, users can have at least one of 4 potential roles [30]—Project Admin, OrgAdmin, Lead, and Member—each with distinct responsibilities and associated risks (description in Table S2 in Multimedia Appendix 1, relationships between the roles in Figure 1). For high-risk data or models, individuals who will be assigned to critical roles must be highly trusted and reviewed (Table 2, impacts and recommendations for vetting described in Table S2 in Multimedia Appendix 1).

Complicating the relationships between the roles is governmental oversight. The Department of Justice’s newly created data security program that is based on an executive order to prevent the sharing of US bulk data with Countries of Concern (CoCs) [31] was launched recently [32] (28 CFR Part 202). This recently escalated to barring CoCs from access to National Institutes of Health databases [33]. Human genomic data, a likely data type to be included in datasets used in FL by AMCs, is covered under “human omic data and associated biospecimens,” one of the 6 categories of sensitive personal data. With FL, the data does not move. However, there is a potential vulnerability if the lead role is a “bad actor” or just careless and implements a high-capacity large language model that memorizes the data [34]. This could allow the recovery of the training data via targeted prompting [35]. While this is a clear privacy concern, there are additional ramifications, as the executive order impacts not just entities associated with CoCs but also individuals who reside in a CoC or are employed by entities within a given CoC. This highlights the importance of having a formal mechanism for reviewing the individuals granted each role, especially for higher risk data as well as the model itself.

NVIDIA FLARE provides a mechanism to fine-tune privileges within the roles through authorization policies, which allows each institution (site) to enforce their own access requirements. Authorization policies are carefully reviewed by the CISO, who works closely with the AIGC to ensure alignment between security and operational requirements. The general recommendation for projects of all risks is to follow the design principle of “least privilege” [36], that is, providing the minimal amount of access required to perform a given role with respect to both the model training process and the data. Restricted role authorization would be seen as necessary for any high-risk data (this would be unaffected by model risk), but application to both low- and high-risk datasets would not place an undue burden on the sites.

One area that is likely to be of contention for model training is support for BYOC or “bring-your-own-code.” This is a good example of the difficulty balancing the responsibility of the Prime Site Lead (lead at site that developed model) to protect the model in terms of both performance (including fairness) as well as security and the responsibility of the external OrgAdmin(s) to protect the data or system of their site. Specifically, custom code from the Prime Site Lead can be perceived as a potential threat to both the external sites and hosted data by the OrgAdmins. When the Prime Site Lead needs to make an update, and BYOC is not enabled, they are dependent upon the OrgAdmin or other system administrators at the external sites to implement those changes. Importantly, a compromised client at the external site would have access to the code. This has several implications: first, any confidential code could be accessed, jeopardizing relevant intellectual property; additionally, the integrity of the code could be compromised. For instance, the code could be modified to attack the model, allowing data extraction or performance degradation.

Privacy policies implement additional protections and can be implemented separately for each site [37]. They are designed to thwart so-called feature-inference or reconstruction attacks [9], which attempt to recover the client’s training data, given the updates obtained by the server. In particular, this is where privacy-preserving filters such as DP are implemented [38]. DP is used in FL to lower the probability that patient-level information is revealed through model updates, most notably in sensitive domains like genomics [39] and EHR analyses. While DP offers mathematical privacy guarantees, practical implementation requires careful selection of privacy budgets and recognition of real trade-offs in model utility. DP must be governance-reviewed and considered alongside other protections, given its clear limitations in the federated paradigm. One DP implementation available in NVIDIA FLARE involves the specification of three main parameters: *eps1*, *eps3,* and fraction of the model to upload [38]. The main challenge is the choice of *eps* values as they can be dataset or site specific [40] and impact performance. This was highlighted in an evaluation that used the electronic intensive care unit Collaborative Research Database [41]. The authors found it difficult to achieve good performance for DP (both for hyperparameters that were fixed globally or chosen at each site). This well-known trade-off between security and performance [42] is also observed in benchmarking using this DP implementation [38].

To make the determination of the *eps* values tractable, we suggest that the DP approach be parameterized by first conducting a smaller-scale study with lower-risk data to evaluate the DP parameters with respect to the model performance measures, for instance, using similar publicly available data from Genomic Data Commons [43] or one of the other Cancer Research Data Commons resources [44]. The lowest viable *eps* values would then be chosen from the smaller-scale study. If this is not the case using acceptably low values of DP, then the Prime Site Lead can reevaluate the model, the need for access to sensitive data or FL as a viable strategy. Also, as a guide, federated DP has been evaluated in settings relevant to AMCs such as genomics [45-48].

In addition to DP, NVIDIA FLARE provides an implementation of another potentially complementary tool for reducing data risk to AMCs, called HE [49]. HE works in addition to standard security provided by Secure Sockets Layer and allows each client to further specially encrypt each model update such that the server can still aggregate them [50]. Importantly, the server never has access to the unencrypted updates. The client, however, can decrypt the received model and can continue training on its local data. In this manner, HE increases data security with minimal impacts on model performance and can protect against attempts to recover client data from model updates. However, the extra encryption steps do result in increased time needed for an FL training run. For instance, in benchmarks from NVIDIA, a 20% increase in time was noted along with a 15× increase in message size [49]. This addresses the risk of insider attacks on server infrastructure hosting FL but comes with substantial computational and resource costs. Its application in AMC settings is highly specialized, suitable mainly for high-sensitivity projects and should be governance-reviewed rather than adopted as standard practice. Note that HE does not protect against private information being memorized in the final model as part of the training process and therefore could also be considered in conjunction with a privacy-preserving approach such as DP [12]. Due to the complexity of DP and HE and related approaches that are under development, it is vital that staff members who are versed in their use and performance tradeoffs are consulted when implementing methods to enhance security and privacy.

Throughout the entire process, effective collaboration between the CISO and AIGC teams is essential. The CISO provides leadership on technical security, compliance, and risk mitigation, while the AIGC brings expertise in ethical, operational, and clinical considerations. Together, they create a comprehensive, enforceable governance structure that balances innovation with safety, privacy, and regulatory compliance. This partnership is crucial for ensuring that FL initiatives at AMCs are both secure and aligned with the institution’s mission and values.

## Operationalizing the Framework: Illustrative Examples

In order to demonstrate how this framework could be operationalized, we will highlight 2 examples. In our first example, there is a collaboration among multiple AMCs to develop an extreme gradient boosting model using deidentified clinical data for research use only purposes. As the data are deidentified, they would be considered low risk; additionally, since the model has limited capacity and would be used only in a research context, it would also be considered low risk. Only an expedited AIGR would be needed in this scenario with standard role-based access control. On the other hand, if genomic data were also used, the data could be considered high risk depending on the institutional (and national) policies while the model would continue to be considered low risk. In this scenario, an AIGR and SecR would be necessary to ensure that the data were protected even if used with a low-risk model. The necessary artifacts here would be literature documentation and computational notebooks with analyses, indicating lack of memorization and robustness to attack for the extreme gradient boosting model. Similarly, role qualification documentation and the “authorization.json” config file for NVIDIA FLARE would be needed to ensure appropriate access to the data and model.

As a second example, a group of AMCs is collaborating to develop a large language model to be used as part of clinical decision support using electronic health record data—including clinical genomics. This would be a scenario where both the data and model would be considered high risk, especially given its potential use operationally in a clinical setting. In this case, a thorough AIGR and SecR would be needed, and the implementation of additional privacy and security measures such as HE and DP would be required. The full range of artifacts presented in Textbox 1 would need to be collected and reviewed by both AIGC and the office of the CISO.

## Discussion

FL holds tremendous promise for high-risk data sharing and model development in AMCs. Our proposed risk framework for AMCs assists CISOs and AIGC as well as other leadership by providing 4 main categories based on data and model risk, ensuring they are clearly communicated and transparent. This is critical given the rapidly evolving US regulatory developments around AI.

In addition, there is significant movement internationally toward proportionate governance of AI in health care. The European Union’s Artificial Intelligence Act, which came into force in August 2024, classifies health care AI applications into divergent risk categories (eg, high risk: clinical decision support, and low risk: AI chatbots providing advice on well-being) [51]. High-risk AI in health care must meet strict requirements for risk management, data governance, human oversight (eg, clinicians must be able to contest AI outputs), transparency, and bias auditing, with integrated conformity assessments for both safety and data protection. In parallel, Canada’s Artificial Intelligence and Data Act applies a risk-based framework to “high-impact” AI systems, focusing on protecting health, safety, and fundamental rights. The Artificial Intelligence and Data Act emphasizes robust risk assessment and mitigation, data management integrity, and continuous monitoring to ensure fairness and prevent harm or bias in health care applications. These initiatives demonstrate an emerging global consensus: international policy is converging on standards-informed and risk-proportionate oversight for AI in health. FL leads to shared legal responsibilities that need to be clearly understood based on data and model risks [52]. The work presented here is timely and can also support these efforts by allowing us to move from policy and regulation to implementation.

This work can also help guide operational strategies. For example, the use of privacy and security technologies such as DP and HE should be limited to high-risk data due to their costs, whether in terms of lower performance for DP or increased resource utilization, such as HE. In addition, there are hardware-based alternatives that can be considered such as Trusted Execution Environments as well as other approaches to confidential computing. NVIDIA FLARE can leverage Trusted Execution Environments as part of a confidential computing strategy to provide stronger security and privacy guarantees for FL. NVIDIA FLARE is adding support for virtual machine-based confidential computing technologies for both central processing units and graphics processing units, enabling further protection against compromised clients [53,54].

AI governance should play a key role in mitigating model risk by requiring certification of models before they are to be deployed on an FL network. This would help ensure that the models being developed are effective, fair, and preserve privacy. However, current levels of heterogeneity in AI maturity models and governance have implications for FL partnerships if the prime site does not have the appropriate infrastructure in place to support the required activities (including pretraining certification, etc). For consortiums, the best approach may be a centralized AI governance process with the most mature site leading the evaluation. The establishment of consensus benchmarking metrics for security and privacy review (as proposed in the former executive order 14110 [55]) would also help facilitate this process tremendously. Security and risk management in FL, as with most other complex systems, is a continually evolving landscape of newly identified risks and countermeasures. For instance, even with the implementation of DP and HE, there are still cases where information can be extracted from an FL system [56]. Importantly, attacks such as this require insider knowledge of the system and problem domain. We acknowledge that FL is an evolving domain, and as it becomes increasingly adopted by AMCs, guidance will need to be updated due to improved methodologies and to address new challenges.

## Supplementary material

10.2196/80022 Multimedia Appendix 1 Listing of federated learning platforms, which provides an overview and security considerations for the NVIDIA Federated Learning Application Runtime Environment user roles.

## Abbreviations

AI: artificial intelligence
AIGC: Artificial Intelligence Governance Committee
AIGR: artificial intelligence governance review
AMC: academic medical center
CISO: Chief Information Security Officer
CoC: Countries of Concern
DP: differential privacy
FL: federated learning
HE: homomorphic encryption
IEC: International Electrotechnical Commission
ISO: International Organization for Standardization
ML: machine learning
NIST AI RMF: National Institute of Standards and Technology Artificial Intelligence Risk Management Framework
NVIDIA FLARE: NVIDIA Federated Learning Application Runtime Environment
SecR: security review
